# Do the disc degeneration and osteophyte contribute to the curve rigidity of degenerative scoliosis?

**DOI:** 10.1186/s12891-017-1471-y

**Published:** 2017-03-29

**Authors:** Feng Zhu, Hongda Bao, Peng Yan, Shunan Liu, Mike Bao, Zezhang Zhu, Zhen Liu, Yong Qiu

**Affiliations:** 1 Spine Surgery, The Affiliated Drum Tower Hospital of Nanjing University Medical School, Zhongshan Road 321, Nanjing, 210008 China; 2 Department of Orthopaedics & Traumatology, The Chinese University of Hong Kong, Hong Kong, China; 3 Joint Scoliosis Research Centre of the Chinese University of Hong Kong and Nanjing University, Nanjing, China; 4 Geisel School of Medicine, Dartmouth College, Hanover, NH USA

**Keywords:** Disc degeneration, Osteophyte, Curve flexibility, Degenerative scoliosis

## Abstract

**Background:**

The factors associated with lateral curve flexibility in degenerative scoliosis have not been well documented. Disc degeneration could result in significant change in stiffness and range of motion in lateral bending films. The osteophytes could be commonly observed in degenerative spine but the relationship between osteophyte formation and curve flexibility remains controversial. The aim of the current study is to clarify if the disc degeneration and osteophyte formation were both associated with curve flexibility of degenerative scoliosis.

**Methods:**

A total of 85 patients were retrospectively analyzed. The inclusion criteria were as follow: age greater than 45 years, diagnosed as degenerative scoliosis and coronal Cobb angle greater than 20°. Curve flexibility was calculated based on Cobb angle, and range of motion (ROM) was based on disc angle evaluation. Regional disc degeneration score (RDS) was obtained according to Pfirrmann classification and osteophyte formation score (OFS) was based on Nanthan classification. Spearman correlation was performed to analyze the relationship between curve flexibility and RDS as well as OFS.

**Results:**

Moderate correlation was found between RDS and curve flexibility with a Spearman coefficient of −0.487 (*P* = 0.009). Similarly, moderate correlation was observed between curve flexibility and OFS with a Spearman coefficient of −0.429 (*P* = 0.012). Strong correlation was found between apical ROM and OFS compared to the relationship between curve flexibility and OFS with a Spearman coefficient of −0.627 (*P* < 0.001).

**Conclusions:**

Both disc degeneration and osteophytes formation correlated with curve rigidity. The pre-operative evaluation of both features may aid in the surgical decision-making in degenerative scoliosis patients.

## Background

The evaluation of spinal lateral flexibility on bending films is vital for classifying structural curve, determining fusion levels and even predicting correction outcome. Several factors have been proposed to be associated with curve flexibility in idiopathic scoliosis, including age, curve magnitude and location [1]. Similarly, in degenerative scoliosis, the lateral flexibility also helps to determine whether the asymmetric osteotomy should be performed and to determine the upper and lower instrumented vertebra. However, the factors associated with lateral curve flexibility in degenerative scoliosis have not been well documented.

The intervertebral disc serves as shock absorber in spine and allows for mobility of the spine. As aging of the spine, the loss of aggrecan lowers the ability of resistance to longitudinal compression during daily activities, resulting in reduced disc height and altered mechanical properties of the disc [2]. For degenerative scoliosis, specifically, a consensus has been reached that it was asymmetric disc degeneration that triggers coronal curvature of spine. Disc is the main load-bearing structure and functional motion elements, maintaining the stability and flexibility of spine together with posterior elements such as facet joints [3, 4]. Zirbel et al. [5] investigated disc degeneration based on functional spinal unit under physiological conditions (including a compressive follower load and at body temperature) and found that disc degeneration resulted in significant change in stiffness and range of motion in lateral bending films. Homminga et al. [6] also stressed in a finite element study that disc degeneration compromised the stabilizing mechanisms of the elderly spine.

As degeneration of spine progresses, the osteophytes could also be commonly observed. According to Yasuda et al. [7], the osteophyte formation may help provide stabilization for wedging segments to offset the instability in degenerative scoliosis. However, some other studies reported that asymmetric osteophytes may increase the incidence of degenerative scoliosis, which is mainly the presentation of spinal instability [8]. Thus the relationship between osteophyte formation and curve flexibility in degenerative scoliosis need further study. The aim of the current study is to clarify if the disc degeneration and osteophyte formation were both associated with curve flexibility of degenerative scoliosis.

## Methods

### Subjects

This study was a retrospective review of 85 patients with degenerative scoliosis (11 male and 74 female). The age of patients averaged 59.26 ± 7.81 years old (45–76 years old) and Cobb angle averaged 38.17 ± 15.27° (20–51°). The inclusion criteria were as follow: age greater than 45 years, diagnosed as degenerative scoliosis (criteria according to Iida et al. [9]), coronal Cobb angle greater than 20° and availability of long-cassette standing upright coronal radiographs of spine, lateral bending radiographs as well as lumbar magnetic resonance images (MRI). Patients with prior spine surgery, spinal tumors, isthmic spondylolisthesis, spinal tuberculosis or osteoporotic fracture were excluded from the study. The study was approved by the clinical research ethics committee of our hospital.

### Radiographic measurements

All radiographical parameters were measured using Surgimap Software (Version: 2.0.8; Nemaris Inc., New York, NY). Cobb angle was obtained both on long-cassette standing upright coronal radiographs and supine lateral-bending radiographs. In degenerative scoliosis patients, the flexibility is evaluated by using bending film toward convex side. Pushing force was applied on the apex of the curve from the convex side with the maximum strength when taking the bending films. The change of curvature severity could be used to evaluate the flexibility of the curve. Curve flexibility was then calculated with the following formula:

$$ \mathrm{Flexibility}=\frac{Cobb\  angle\  of\  standing\  position\mathit{\hbox{-}} Cobb\  angle\  of\  bending\  position}{Cobb\  angle\  of\  standing\  position} $$

The range of motion (ROM) of each disc level was also measured, defined as follow:

$$ \mathrm{ROM}=\frac{disc\  angle\kern0.15em  of\; standing\kern0.15em  position\mathit{\hbox{-}} disc\; angle\; of\; bending\; position}{disc\kern0.37em  angle\; of\; standing\; position} $$

**Fig. 1:**
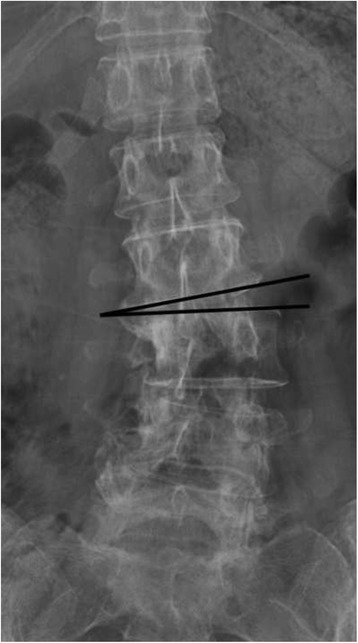
Illustration of measurement of disc angle

Only the ROM of disc located 2 levels above and below apex was analyzed. Disc angle was defined as the angle between the superior and inferior endplate of the corresponding disc level (Fig. 1).

**Fig. 2:**
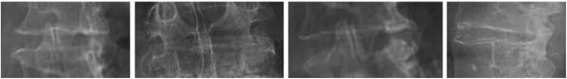
Illustration of Nathan classification for osteophyte according to Yasuda et al. [7]

Osteophyte formation was evaluated on long-cassette standing upright coronal radiographs using Nathan classification [7, 10] (Fig. 2): Grade I osteophytes only appear as isolated points of initial hyperostosis. Grade II osteophytes are bone protrusions projecting more or less horizontally from the vertebral body. Grade III osteophytes assume the characteristic shape of a bird’s beak shape and come into close contact with the free ends of the osteophytes on the adjacent vertebra. Grade IV osteophytes occur, when the osteophytes of the 2 adjacent vertebrae are fused together. For each curve, osteophyte formation score (OFS) was obtained based on the grade of apical osteophyte: 1 score for Grade I osteophyte and 4 score for Grade IV osteophyte.

**Fig. 3:**
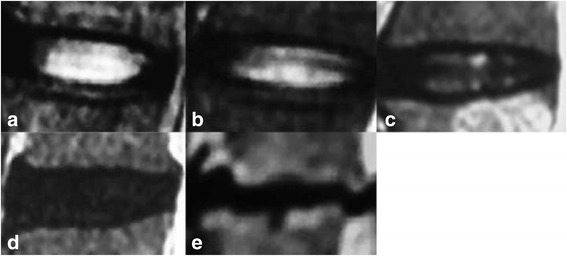
The Pfirrmann score system for assessing lumbar disc degeneration. A, Grade I, homogeneous disc with bright hyperintense white signal intensity and a normal disc height. B, Grade II, inhomogeneous disc with a hyperintense white signal. The distinction between the nucleus pulposus and annulus fibrosus is clear, and the disc height is normal, with or without horizontal gray bands. C, Grade III, inhomogeneous disc with intermediate gray signal intensity. The distinction between the nucleus pulposus and annulus fibrosus is unclear, and the disc height is normal or slightly decreased. D, Grade IV, inhomogeneous disc with hypointense dark gray signal intensity. The distinction between the nucleus pulposus and annulus fibrosus is lost, and the disc height is normal or moderately decreased. E, Grade V, inhomogeneous disc with a hypointense black signal intensity. The distinction between the nucleus pulposus and annulus fibrosus is lost, and the disc space is collapsed

The lumbar MRI between L1 and S1 was performed with a 1.5-T MRI system (GyroscanIntera; Philips Medical Systems, Best, The Netherlands). Based on Pfirrmann disc degeneration classification [11] (Fig. 3, Grade I, homogeneous disc with bright hyperintense white signal intensity and a normal disc height. Grade II, inhomogeneous disc with a hyperintense white signal. Grade III, inhomogeneous disc with intermediate gray signal intensity. Grade IV, inhomogeneous disc with hypointense dark gray signal intensity. Grade V, inhomogeneous disc with a hypointense black signal intensity, the disc space is collapsed.), 5 grades were assigned to sagittal T2-weighted images, representing a progression from normal disc to severe disc degeneration, where grade I corresponded to no degeneration while grade V represented the most severe degeneration. Scoring was calculated for convenient assessment, to grade I, 1 score was given, while for grade V, 5 score was given. Lower scores represented more favorable disc conditions. The sum of the disc degeneration score from L1-L2 to L5-S1 was calculated and defined as the regional lumbar disc score (RDS). The apical disc was defined as the disc inferior to apical vertebra when the apex located at vertebra.

### Statistical analysis

Data were analyzed using SPSS statistical software (SPSS 20.0, SPSS Inc., Chicago, IL). Summary statistics from analysis of variance calculations were used to provide 95% prediction limits for measurement error. The correlation was analyzed by the Spearman correlation coefficient. For absolute values of correlation coefficient, 0–0.19 is regarded as very weak, 0.2–0.39 as weak, 0.40–0.59 as moderate, 0.6–0.79 as strong and 0.8–1 as very strong correlation [12]. Significance was defined at *P* < 0.05.

## Results

The age of the patients averaged 59.26 ± 7.81 years old (45–76 years old). The average main curve magnitude was 38.17 ± 15.27° (range, 20–51°) on standing films and 24.61 ± 14.85° (range, 14–40°) on lateral bending films, making the main curve flexibility 35.52% ± 13.72 (Table 1). After surgical correction, the main curve magnitude decreased to 17.73 ± 8.34° and the correction rate averaged 53.54% ± 10.61.

**Table 1:** Demographics of the cohort

	Mean	SD	Range
Age (years)	59.26	7.81	45–76
Gender			
Male	11		
Female	74		
Baseline standing Cobb angle (°)	38.17	15.27	20.04–50.86
Baseline Bending Cobb angle (°)	24.61	14.85	14.42–40.31
Flexibility (%)	35.52	13.72	12.57–46.24
Post-op Cobb angle (°)	17.73	8.34	8.27–26.44

A total of 392 discs were retrospectively analyzed, of which 10 were grade I, 82 grade II, 93 grade III, 126 grade IV and 81 grade V. The average RDS was 3.48 ± 0.61. Moderate correlation was found between RDS and curve flexibility with a Spearman coefficient of −0.487 (*P* = 0.009). Regarding to the relationship between segmental disc degeneration score and ROM, the negative correlations between ROM and disc degeneration score were significant when only including apical discs (Table 2). However, only weak correlation was found between ROM and disc degeneration in all 392 discs (apical discs and end vertebral discs) with a coefficient of −0.261 (*P* = 0.084).

**Table 2:** Relationship between apical disc degeneration and apical ROM

Location	Degeneration score	Disc angle on standing films	Disc angle on bending films	ROM	*r*	*P*
Apical disc −1	2.56 ± 0.63	5.50 ± 2.47	3.10 ± 1.96	43.64 ± 23.72	−0.530	<0.001*
Apical disc	4.23 ± 0.71	10.12 ± 3.17	8.11 ± 3.25	19.86 ± 14.88	−0.527	<0.001*
Apical disc +1	3.34 ± 0.66	6.42 ± 2.81	4.78 ± 2.13	25.54 ± 16.11	−0.394	0.015*
Apical disc +2	2.92 ± 0.68	4.75 ± 2.49	2.89 ± 1.77	39.16 ± 16.54	−0.425	0.012*

*P < 0.05

**Fig. 4:**
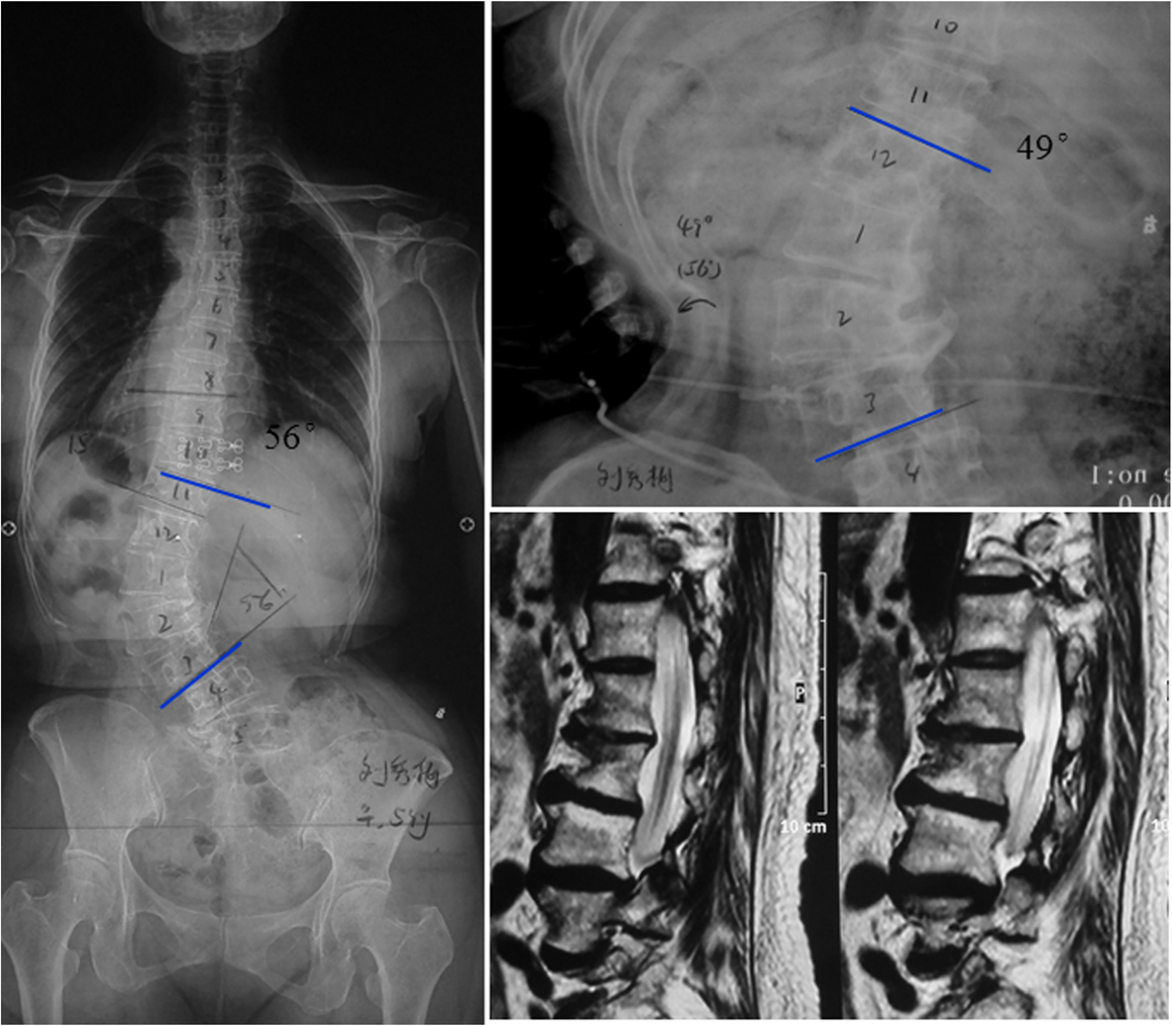
59 years old female patient with degenerative scoliosis. Lumbar Cobb angle was 56° on standing whole spine film. On supine lateral bending film, lumbar Cobb angle decreased to 49°, with a correction rate of only 12.5%. Lumbar MRI showed severe disc degeneration; Grade IV osteophyte could be found on concave side of the curvature

Regarding to osteophyte formation, similarly, moderate correlation was observed between curve flexibility and OFS with a Spearman coefficient of −0.429 (*P* = 0.012). Strong correlation was found between apical ROM and OFS with a Spearman coefficient of −0.627 (*P* < 0.001) compared to the relationship between curve flexibility and OFS. The relationship between osteophyte and disc degeneration was also analyzed. OFS was found to significantly correlate to both RDS and apical degeneration score (*r* = 0.381 and 0.646, *P* = 0.021 and <0.001, respectively, Fig. 4).

## Discussion

Curve rigidity is one of the vital radiographic parameters for pre-operative evaluation during surgical decision-making [13, 14]. For example, fusion levels could be decided with the help of flexibility evaluation [15]. In degenerative scoliosis, the grade of osteotomy could also be determined by assessment of curve rigidity. In patients with rigid curve, utilization of 3-column osteotomy is indicated; otherwise SPO osteotomy may just fit for the scenario of high flexibility [16]. Therefore, curve flexibility and its affecting factors in degenerative scoliosis should be further investigated. The current study stressed that curve flexibility in degenerative scoliosis was correlated with both disc degeneration and osteophyte formation, making the disc degeneration and osteophyte the supplemental factors for decision of fusion levels.

The relationship between disc degeneration and spinal instability has been reported in many biomechanical studies. Mimura et al. [17] reported a significantly decrease in ROM due to disc degeneration in lateral bending position. Costi et al. [18] investigated the effect of disc degeneration on disc mechanics of functional spine unite, and found significant increases in stiffness with the condition of disc degeneration. The results of the present study also supported previous conclusions through stressing correlation between RDS and curve flexibility in degenerative scoliosis patient cohort. However, an unexpected finding was a weak correlation between ROM and disc degeneration at each disc levels. Zirbel et al. [5] found in a biomechanical study that the relationship between curve stiffness and ROM was not linear, generally due to the inhomogeneous histologic features of disc at different degeneration grades. According to the hypothesis proposed by Kirkaldy-Willis [19], grade IV disc degeneration was the most unstable condition with severe degeneration but well-preserved disc height. When the disc degeneration continues, collapse of disc will occur, defining as grade V degeneration, and the disc will become re-stabled. That means grade IV disc has the largest ROM while grade V disc is with less ROM. This may explain why we did not observe moderate to strong correlation between ROM and the disc score with all 392 discs.

More specifically, the ROM of apical disc levels was analyzed. At the two disc levels cranial and caudal to apex respectively, negative correlations between ROM and disc degeneration were all determined as significant. Apical discs were with more severe disc degeneration and lower ROM compared to the other two discs above and below apex. Based on clinical experience, apical region is the most rigid part of curve, especially in degenerative scoliosis. The ROM of disc analyzed in the present study could also be regarded as the segmental flexibility of lumbar curve. Since the curve flexibility was the accumulation of segmental flexibility, rigid apical region took more responsibility for decreased curve flexibility. This speculation was in accordance with the degenerative cascade triggering degenerative scoliosis: initial disc wedging started, followed by wedged disc degeneration, this triggering disc then became apical disc with most severe degeneration [20, 21].

It has been reported that osteophyte formation provided restablization for wedging segments in degenerative scoliosis [7]. In addition, a cadaveric study also showed that osteophyte formation resisted lateral bending movements [22]. In the present study, osteophyte formation significantly correlated with decreased curve flexibility, indicating that patients with severe osteophyte formation should receive more powerful osteotomy such as PSO. Nathan et al. [10] developed a classification for osteophytes, in which Grade IV osteophyte was defined as fusion of osteophytes between 2 adjacent vertebra. Grade IV osteophyte, or more generally all grades of osteophytes on the concavity of the curve, resembled bony bridge in congenital scoliosis due to unsegementation. In congenital scoliosis, bony bridge always represented decreased flexibility and rigid curve. Therefore, osteophytes formation could be regarded as a possible method allowing degenerative scoliosis to stabilize the spine. Interestingly, Jimbo et al. [8] proposed in a prospective study that unilateral osteophyte formation, defined as asymmetric osteophyte with 5 mm difference between convex and concave side, was risk factor for curve progression in degenerative scoliosis, which was a sign of instability. Our results did not conflict with their conclusions. Larger osteophytes always occurred at the concave side of the curve and thus were more likely to fuse as bony bridge, an obvious cause to curve progression, just as the unsegmented vertebra in congenital scoliosis.

Our data revealed the significant correlation between OFS and apical degeneration as well as RDS. Osteophyte formation has been associated with disc degeneration and endplate sclerosis. Animal models recorded that scalpel-induced disc degeneration causes osteophytes to grow in adjacent vertebrae, indicating osteophytes arose from proliferating annulus tissue [23]. Nathan et al. [24] suggested that osteophytes could also arise from tissues including longitudinal ligaments and periosteum. Taken together, disc degeneration and osteophytes formation may be the two sides of one coin and contributed together to deceased curve flexibility.

For degenerative scoliosis patients, different osteotomy grade [25] is determined by requirement of sagittal kyphosis correction to ideal spinopelvic harmony, while the choice of asymmetrical three column osteotomy mainly depends on surgeon’s personal experience without any standardized criteria. The current study demonstrated significant correlations between curve flexibility and disc degeneration as well as osteophyte, indicating the possibility to establish a disc and osteophyte based grading system to determine the necessity of asymmetrical 3 column osteotomy. Further prospective-design study is warranted to establish such grading system.

The curvature severity was not taken into consideration in the current study due to small lumbar curve in this cohort and the etiology. When considering rigidity, curve severity plays a more important role in adolescent idiopathic scoliosis compared to degenerative scoliosis since degenerative scoliosis is characterized with smaller coronal curvature, less rotation but more loss of lumbar lordosis. In addition, previous literature also conformed the association between lumbar coronal curvature and disc degeneration severity [3]. The rigidity of facets is difficult to evaluate with the current technique. The classification of facets degeneration with CT images is also not reliable in degenerative scoliosis due to rotation and deviation of each vertebra. Regarding rotation, it is not as obvious in degenerative scoliosis as it is in idiopathic scoliosis, and thus it can be tolerated without analyzing rotation. The influence of age is also quite different to measure since degenerative scoliosis, as well as disc degenerative and osteophytes formation are the results of aging.

Limitations of this study included the lack of association between curve rigidity and surgical correction, the lack of age-matched normal controls and the lack of consideration of facets degeneration as well as rotation. In addition, this study was conducted as a cross-sectional study, lacking of continuous observation of association between disc degeneration, osteophytes formation and curve flexibility during the progression of spinal degeneration.

## Conclusions

Both disc degeneration and osteophytes formation correlated with curve flexibility. The pre-operative evaluation of both features may aid in the surgical decision-making in degenerative scoliosis patients.

## Abbreviations

OFS: Osteophyte formation score
PSO: Pedicle subtraction osteotomy
RDS: Regional disc degeneration score
ROM: Range of motion
SPO: Smith-peterson osteotomy
